# Medical Titles

**Published:** 1907-06-08

**Authors:** Frederick W. Collingwood

**Affiliations:** Denehurst, Acton Hill, W.


### EDITOR'S LETTER-BOX.

[Our Correspondents are reminded that prolixity is a reat bar to publication, and that brevity of style and conciseness of statemen greatly facilitate early insertion.]

### MEDICAL TITLES.

Sir,—All honour to those who have obtained the M.D. London. Unfortunately, the title of doctor has both a popular and academic significance; the former being well grounded in popular usage.

Further, the public service competitive examinations testify to the high standard of efficiency attained by London licentiates and members. England is inundated with Scotch, Irish, and provincial graduates, M.B.s and M.D.s, all styling themselves doctor, and so it becomes a hardship that English qualified practitioners should be debarred from using the title conferred upon them by conventional usage. Yours truly,

Frederick W. Collingwood
(L.R.C.P.Lond., M.R.C.S.Eng.).

Denehurst, Acton Hill, W., June 1.

